# The effect of resting morphological lip shape during lip movement: A three-dimensional motion analysis study

**DOI:** 10.1016/j.heliyon.2020.e04093

**Published:** 2020-06-01

**Authors:** Siti Hajjar Nasir, Hashmat Popat, Stephen Richmond

**Affiliations:** a Department of Orthodontics, Kulliyyah of Dentistry, International Islamic University Malaysia, Malaysia; b Applied Clinical Research and Public Health Department, School of Dentistry, Cardiff University, United Kingdom

**Keywords:** Anatomy, Lip shape, Lip motion, 3dMD, Lip phenotypes, PCA

## Abstract

**Purpose:**

The aim of this study was to determine the influence of different morphological lip shape during lip movement.

**Method:**

A sample of 80 individuals with three-dimensional facial images at rest and during speech were recorded. Subjects were asked to pronounce four bilabial words in a relaxed manner and scanned using the 3dMDFace™ Dynamic System at 48 frames per second. Six lip landmarks were identified at rest and the landmark displacement vectors for the frame of maximal lip movement for all six visemes were recorded. Principal component analysis was applied to isolate relationship between lip traits and their registered coordinates. Eight specific resting morphological lip traits were identified for each individual. The principal component (PC) scores for each viseme were labelled by lip morphological trait and were graphically visualized as ellipses to discriminate any differences in lip movement.

**Results:**

The first five PCs accounted for up to 95% of the total variance in lip shape during movement, with PC1 accounting for at least 38%. There was no clear discrimination between PC1, PC2 and PC3 for any of the resting morphological lip traits.

**Conclusion:**

Lip shapes during movement are more uniform between individuals and resting morphological lip shape does not influence movement of the lips.

## 1 Introduction

The lips are an important structure in the human face and have been recognized as one of the features that contribute to facial attractiveness. The lips are also involved in expressing various facial expressions such as smiling, pouting and frowning as well as facilitate in swallowing and speech production, particularly when making labial and bilabial sounds [1]. Defects in both anatomy and function of the lips will usually result in poor facial aesthetic, impairment of the speech, drooling and oral spillage.

Evaluation of the soft tissue profile during patient's extra-oral examination are performed regularly. This usually involves an informal assessment of the lips in a relaxed position and on smiling. Because the lips are highly mobile and serve as an important organ for speech production, assessments of lip functions should also be performed. This is particularly important in cleft lip patients, as the aim of lip revision surgery is to restore both the anatomical continuity and function of the lips [2]. Many patients with bilateral cleft lip and palate have muscle deficiencies of the prolabium when assessed at rest. Likewise, unnatural and increased facial asymmetry were also observed during lip movement or facial expressions due to this muscle deficiency problem [3, 4]. Although cleft lip correction surgeries are able to achieve good functional and aesthetic results when evaluated at rest, some patients still showed asymmetry lip movement imbalances and limitation, which seem to be caused by scarring, muscular pull and relatively thinner tissue around the orbicularis oris muscle area [5, 6].

Most research that has investigated the shape and classification of the lips has been carried out using linear measurements of the lip such as lip length, width and fullness [7, 8]. Furthermore, these measurements were carried out using photographs and two-dimensional (2D) radiography of the subjects. Recently, a novel and comprehensive method of classifying the various morphological features of the lips has been identified and this classification provides a detailed description of the lips using a systematic approach [9]. Most of the previously mentioned lips scales and classifications were observed and assessed when the lips are at rest and patient at still.

Numerous methods to quantify lip movement have been reported and ranged from 2D photography to the state-of-the-art three-dimensional (3D) laser scanning and optical stereophotogrammetry. The digital stereophotogrammetric technology system is a reliable tool in the study of facial morphology, growth and the reproduction of facial morphology. A recent systematic review concluded that stereophotogrammetry is sufficiently accurate and reliable for clinical use, with errors below 1 mm [10]. Stereophotogrammetry also offers higher acquisition speed (up to 1.5 milliseconds) and has a high surface coverage (up to 360° of the head) in a single shot [11].

A study by Stavness et al found that anatomical variations of facial muscles are likely to create variations in facial gestures and speech production. These anatomical variations are known to produce differences in speech-specific lip gestures such as lip protrusion and lip rounding [12]. Given that most investigations of lip movements make use of the surface anatomy and the outline of the lips, it will be plausible to assume different resting lip shape or phenotypes will have certain different influence on the movement of the lips during function. Therefore, the purpose of this study was to determine the effect of various resting morphological lip traits during lip movement.

## 2 Materials and methods

### 2.1 Ethics

Ethical approval was obtained previously from South East Wales Research Ethics Committee for the study by Popat et al [13].

### 2.2 Participants

The sample was a participant group of 80 subjects that had been recruited by previous researchers as a control cohort [13]. Subjects were aged between 18 to 40 years with no relevant medical history, no previous facial surgery or facial paralysis, no speech impairment, a full dentition with normal anterior posterior skeletal relationship and British English as their spoken first language. Subjects with facial clefts or syndromes were excluded from this study.

### 2.3 Capturing lip features

The participants were scanned using the 3dMDFace™ Dynamic System (3Q Technologies, Atlanta, GA, USA) at 48 frames per second. The scanner uses six A501kc digital cameras of 1.3 megapixel (MP) resolution. All subjects were orientated into natural head posture and were asked to pronounce four words, which were *puppy*, *rope*, *baby* and *bob*. Subjects were requested to say these words in a normal relaxed manner without any emotions or facial expressions.

### 2.4 Image processing

**Figure 1:**
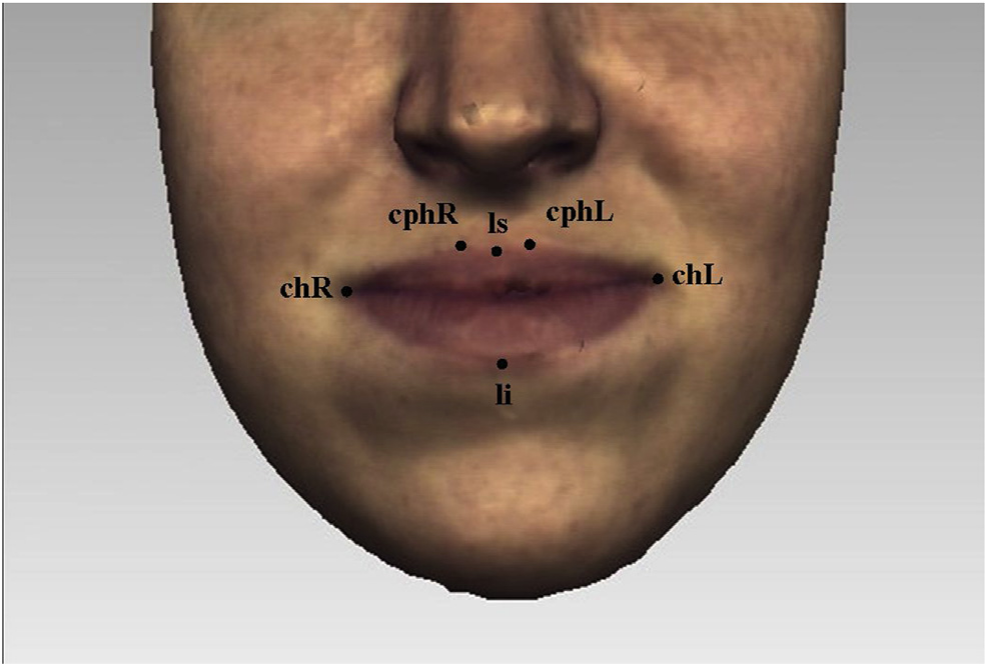
Lip landmarks at rest. *Labiale superius (ls)* - the midpoint of the upper vermilion line; *labiale inferius (li)* – the midpoint of the lower vermilion line; *crista philtri (cph L/R)* – the point on the left and right elevated margins of the philtrum above the vermilion line; *cheilion (ch L/R)* – the point located at the left and right labial commissure.

**Figure 2:**
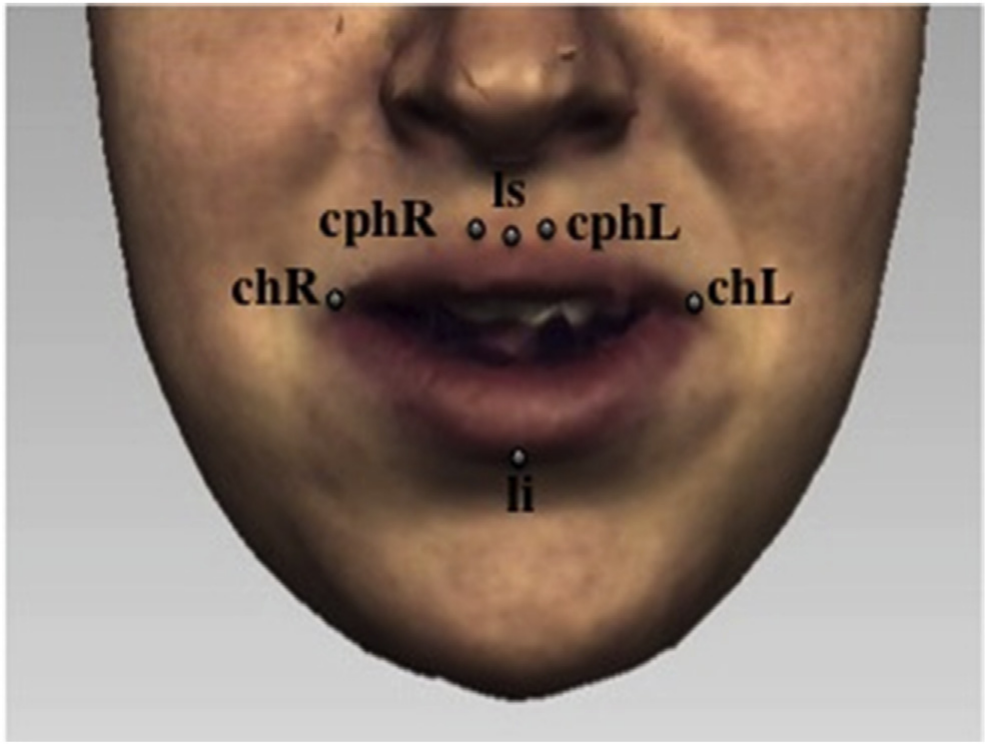
Lip landmarks during maximal displacements.

Six landmarks were placed around the lips for each 3D facial shell (Figure 1). The color texture overlying the 3D facial mesh was maintained during landmark identification. All six lip landmarks were identified at rest and the landmark displacement vectors (x, y, z coordinates) for the frame of maximal lip movement of six visemes (puppy, puppy, baby, baby, rope and bob) were recorded (Figure 2). The frame of maximal lip movement represented the point at which the upper and lower lips were most apart in the vertical plane for the visemes puppy and baby, where the commissures were at their widest for the visemes puppy and baby, and where the lips were at their most protrusive for the visemes rope and bob. This frame was selected by direct observation. Registration and alignment of the coordinates for all landmarks in the dataset were carried out using Generalized Procrustes Analysis (GPA).

### 2.5 Lip traits identification and lip movements association

All subjects lip features at rest were then classified into distinctive types of lip traits according to a modified version of the lip classifications developed by Wilson et al. [9]. The detailed descriptions of lips and the subcategory scores are presented in Table 1. Traits with four or more subcategories were simplified into a maximum of three subcategories to maximize groupings. The association between lips traits and their movements were investigated using PAST software [14]. Subjects with similar lip traits and their landmark coordinates for each of the visemes were grouped together.

**Table 1:** Modified lip traits classification adapted from Wilson et al [9].

Lip Traits	Description & score
Nasiolabial angle (NLA)	0 – Acute
	1 – Normal
	2 - Obtuse
Philtrum shape (PHS)	0 – Smooth/shallow philtrum
	1 – Average philtrum
	2 – Deep groove philtrum
Philtrum width (PHW)	0 – Narrow
	1 – Average
	2 – Wide
Cupid's bow shape (CBS)	0 – Flat
	1 – U-shaped
	2 – Sharp v shape
Upper lip vermillion fullness (ULVF)	0 – Thin
	1 – Medium
	2 – Thick
Lower lip vermillion fullness (LLVS)	0 – Thin
	1 – Medium
	2 – Thick
Lip commissures (LC)	0 – Upturned
	1 – Straight
	2 – Downturned
Lower lip tone (LLT)	0 – None/slight muscle tonicity
	1 – Moderate muscle tonicity
	2 – Marked muscle tonicity

### 2.6 Statistical analysis

Principal component analysis (PCA) was used in this study to isolate the relationship between lip traits and their registered coordinates. This analysis transforms a large number of interrelated variables to a new set of variables or principal components (PCs), and are ordered so that the first PCs (PC1, PC2, PC3 and so on) retain most of the variation present in all of the original variables [15]. PC scores for each viseme were labelled by lip morphological trait and graphically visualized as scatterplots (for the first three PCs) to discriminate any differences in lip movement.

## 3 Results

### 3.1 Principal component analysis

Lip movements during phonation of each visemes were analysed using PCA. Tables 2, 3, 4, 5, 6, and 7 explained the total lip variance in resting lip shape for 6 visemes. For all visemes, five PCs were extracted with an Eigenvalue greater than 1.0. The total variance in lip shape accounted for by these components ranged from 90-95%.

**Table 2:**
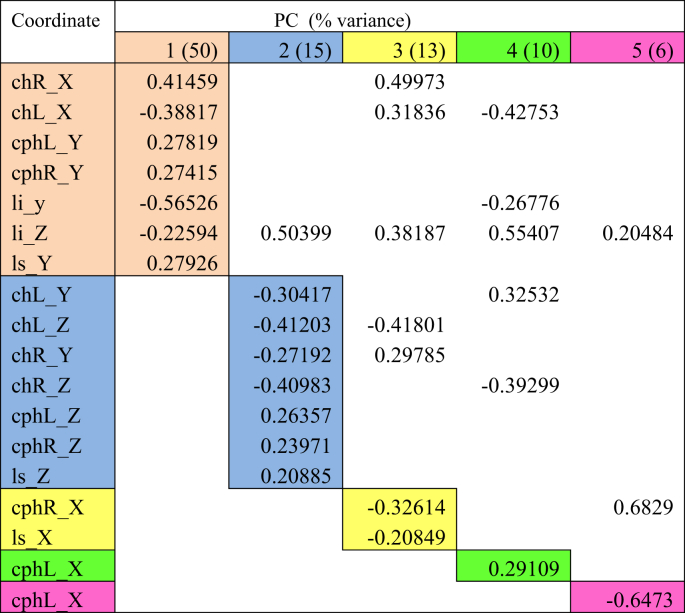
PCA for viseme puppy.

**Table 3:**
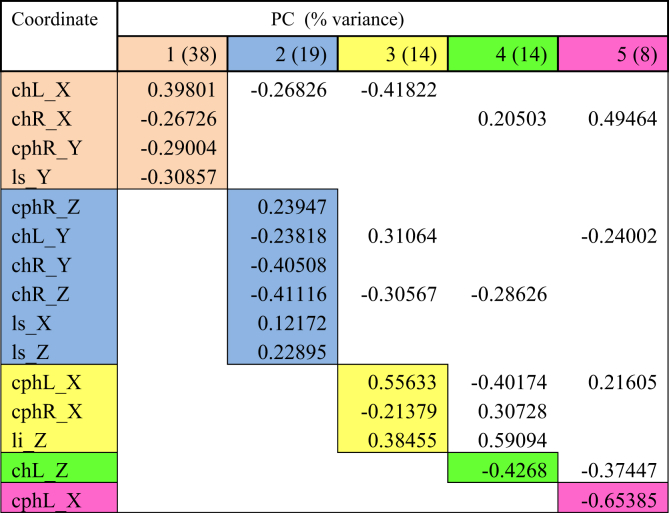
PCA for viseme puppy.

**Table 4:**
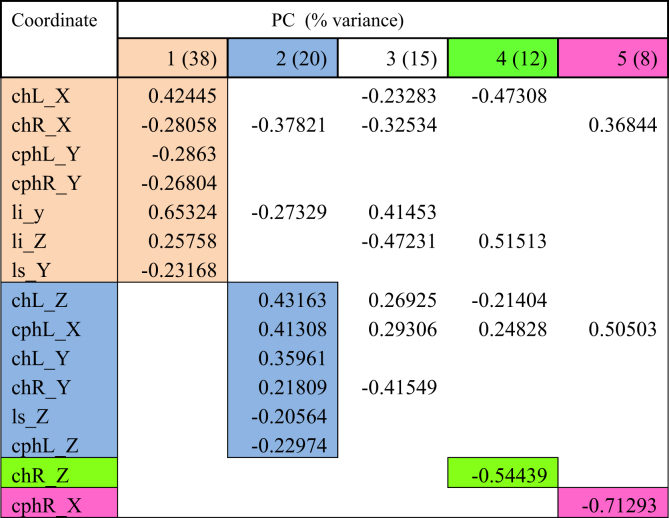
PCA for viseme baby.

**Table 5:**
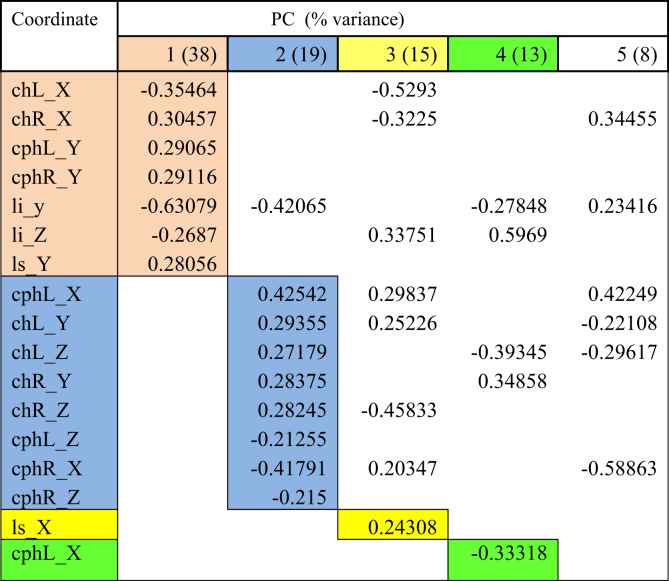
PCA for viseme baby.

**Table 6:**
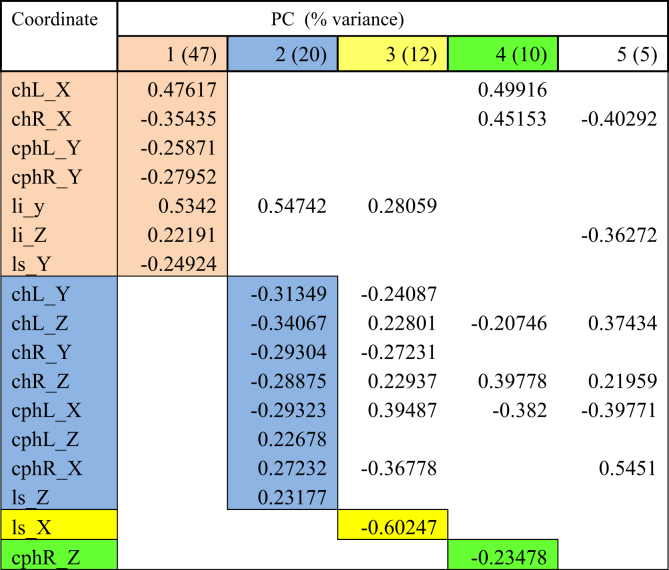
PCA for viseme rope.

**Table 7:**
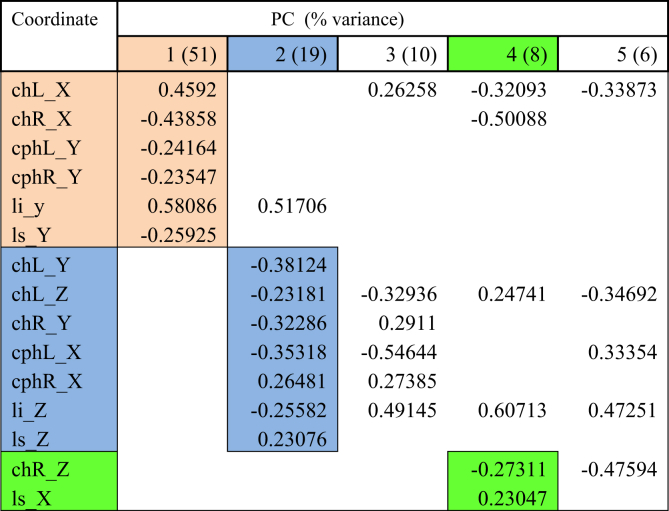
PCA for viseme bob.

The first PC controlled mouth opening and mouth width, with the coordinates *chR X, chL X, cphL Y, cphR Y, li y, li Z, ls Y* loaded on PC1 for all visemes except for viseme puppy. This explained up to 51% of the total variance in lip shape. For puppy, baby, baby and rope, PC1 generally illustrates lateral opening of the upper and lower lip and rounding of the lips with the commissures moving towards each other. Conversely for bob, the mouth opening reduced, and mouth width increased. For puppy, only coordinates *chR X, chL X, cphR Y, ls Y* were loaded on PC1 suggesting more spreading of the lips and commissures, but reduced mouth height as coordinate responsible for movement of the lower lip (*li*) was absent.

PC2 accounted for up to 20% of the total variance of lip shape. The landmarks represented in the second PC mostly involved *z*-coordinates (*chL Z, chR Z, cphL Z, cphR Z, ls Z*) of the upper lip and commissures suggesting protrusive movement of upper lip with the corners of the mouth moving inwards. PC2 also isolated some lateral and upward movement of the upper lip and commissures (*chL Y, chR Y, cphL X, cphR X*) as seen for visemes baby, baby, rope and bob.

PC3 accounted for 13% of the total lip variance and represents a group of transverse coordinates. Landmarks *cphL X* and *cphR X* in puppy displayed loadings with opposite direction implying the widening of philtrum during movement.

**Figure 3:**
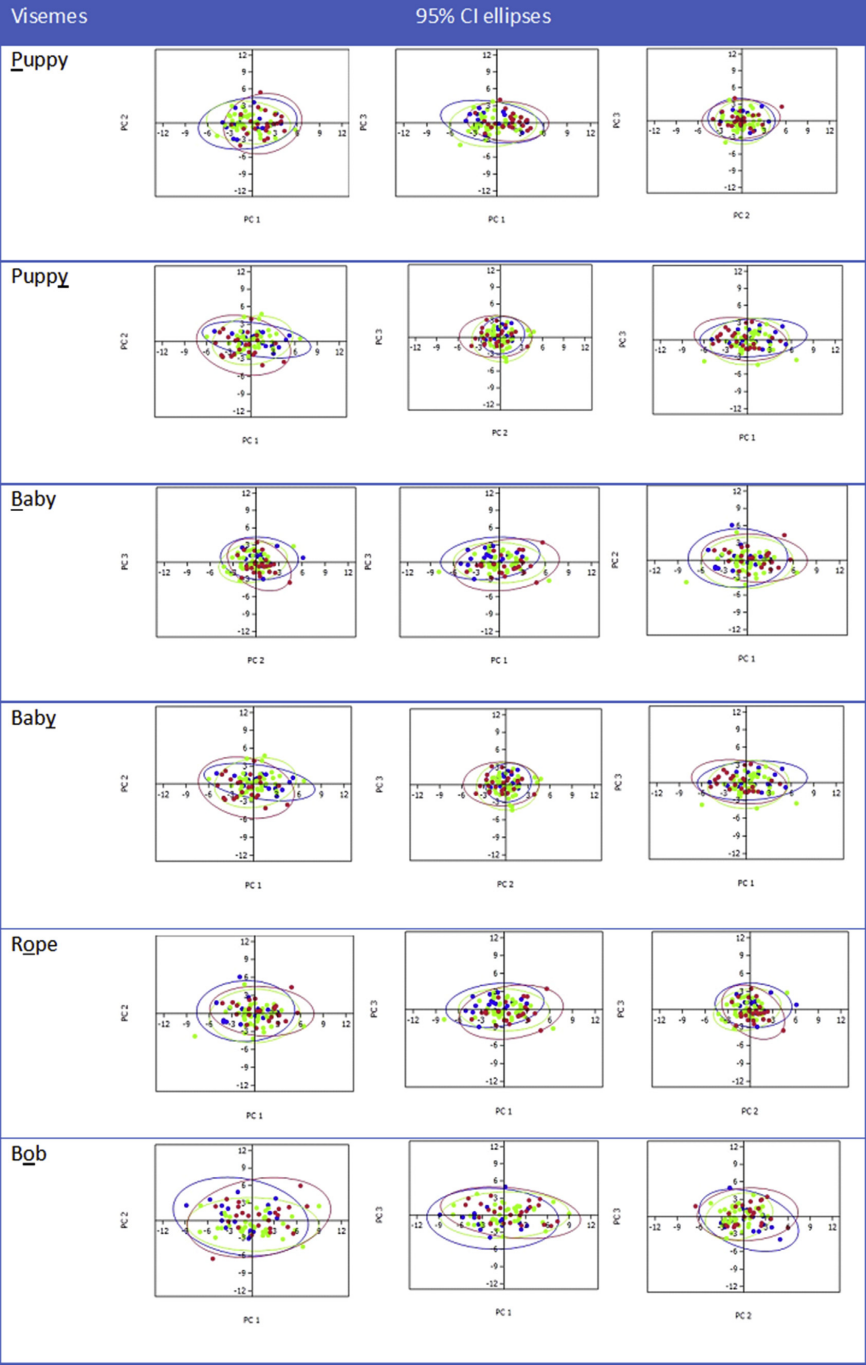
PC1-3 plotted for all visemes by upper lip vermillion fullness; thick (blue), medium (red), thin (green).

PCA was also used to compare the movement of the lip between individual morphological lip traits. PC scores for each lip trait in a category were plotted according to each viseme and any separation of the scores was observed. These scores were calculated using regression coefficients and represent estimates of the scores individual subjects would have received on each of the PCs had they been measured directly. Ellipses encompassing the 95% CI for the PC scores labelled by different lip traits, were plotted which allowed visualisation of which PC (if any) discriminated the traits. Figure 3 showed the first three PCs that were plotted by upper lip vermillion fullness for all six visemes. For PC1-3, the ellipses were overlapping without any obvious separation between thick, medium and thin upper lip vermillion, suggesting that although the fullness of the upper lip varies, there was no difference in the movement of the lips. The analysis for the seven other resting lip morphological traits were performed and showed similar results to the categorization based on upper lip vermillion fullness, i.e. no separation between the 95% confidence intervals (ellipses) indicating no difference in lip movement.

## 4 Discussion

The main objective of this study was to explore the effect of resting lip morphology on lip shape during speech using 3D optical stereophotogrammetry. The relationship between lip traits and their registered coordinates were analyzed using PCA. The main advantage of PCA is data compression, which means that only relevant information is preserved, since the vectors with low variance can be discarded, and thus lessen the dimension of the data set [15]. In this study, PCA enables recognition of apparent and subtle movement of the lips. The first five PCs described up to 95% of the total variance in lip movement for all four words. Majority of the lip movements (rounding and spreading) were explained by PC1 and these vertical (rounding) and transverse (spreading) movements were obvious and expected during lip opening. However, more delicate movements of a specific part of the lip can also be isolated using PCA such as the lateral movement or widening of the philtrum. This is particularly useful when assessing patient with cleft as scarring after surgical correction might inhibit this type of movement. This is in agreement with a recent study that concluded surgical correction of cleft cases had a more restricted upper lip movement as compared to non-cleft volunteers [16]. When evaluating the association between different morphological lip traits during movement, only the first three PCs were measured. They described 72–78% of the total variance in lip movement and no differences were noted between the traits. Although PC4 and PC5 both contributed to the difference in lip movement (explained by eigenvalue more than 1.0), they accounted for a very minimal portion of the whole data (20%) and will not add a significant difference to the result. The result in this study is supported by another study that look into the resting lip shape during speech [17]. They found that there was almost complete overlap of the data from the visemes rope and bob, baby and baby implying that lip movements were very similar between these words.

Most of the investigations that look into the analysis of lip motions reported different measuring tools and methods of assessment. Sforza et al looked at non-verbal facial movements using optoelectronic 3D motion analyzer [18]. In this study, verbal facial gestures were used instead and all four words (*baby, puppy, bob and rope*) were bilabial words, which were performed by bringing both lips together. They represent a range of different lip movements and are words recommended for use in cleft speech assessments [19]. These words incorporate a wide range of lip movements, which include lip opening and lip stretching (*puppy and baby*) as well as lip pursing (*rope and bob*). Previous research has shown that the words puppy and baby are more effective in the assessment of lip movement as compared with smile expressions because they are more reproducible than facial expressions such as smiling [20]. This is because facial expressions frequently involve the action of other facial muscles or action units such as cheek pulling and/or nose wrinkling and also dependent on subject's emotion and response at that particular moment. From a clinical point of view, an objective assessment is important in measuring treatment outcome in patients with cleft lip and palate because the decision for lip revision surgery are usually based upon the presence of disfigurement or asymmetry at rest and an impairment of the lip during function. Hence, the component of emotion should be minimized.

Some changes were made to the lip morphology categories when identifying the lip features in the sample. The modifications were carried out for two main reasons: Firstly, was to reduce and streamline the subcategories or the traits from six to a maximum of three in order to allocate individuals from the sample to distinct subgroups. Given that the sample size was 80 subjects, the allocation rate to each group of 6 traits would be small and irrelevant. Secondly, reducing the subcategories will significantly improve the reliability score between examiners and reducing the error [9]. This is because with 3 subgroups in each category, the distinctions between the groups are greater.

During classification of the lip traits, 2D bitmap (BMP) image files devised from a high-resolution 3D facial shell image were used. These images were capable of accurately displaying and illustrating each subject's lip profiles. Wilson et al compared the surfaces details of images scanned using 3dMD and laser scanner and found that there was a general loss of detail with 3dMD [9]. However, they mentioned that this problem could be overlooked if a simplified version of the categorization were used. Hence, categorization of the lip traits using the 2D BMP image would be reliable and valid.

One of the inclusion criteria for the sample in this study was British English as their spoken first language. This criterion was made because of the well-known influence of language in speech production. Studies have shown that there were specific differences in lip movement during linguistic speech, which suggest that anticipatory labial coarticulation is a learned behavior. A study by Lubker and Gay proposed that speakers of Swedish have more extensive labial protrusion for rounded vowel production as opposed to speakers of American English [21]. Therefore, the sample used here is considered specific to this geographic region and more data from other populations are needed for detailed comparison.

## 5 Conclusion

It can be concluded that whilst resting lip morphology and lip traits are highly variable, lip shapes during movement are more uniform between individuals when compared to resting lip traits. The results from this study can be translated to the clinical setting and provide useful information or baseline data for population comparison, treatment planning process and assessment of treatment outcomes. Moreover, the influence of language during articulation has been recognized to produce different lip mobility. The movements of the lips are mostly dependent on the language itself and their state of emotions or facial expressions during speaking and therefore, can be an area of investigation for future studies.

## Declarations

### Author contribution statement

S. H. Nasir: Conceived and designed the experiments; Performed the experiments; Analyzed and interpreted the data; Wrote the paper.

H. Popat, S. Richmond: Conceived and designed the experiments; Analyzed and interpreted the data; Contributed reagents, materials, analysis tools or data; Wrote the paper.

### Funding statement

This research did not receive any specific grant from funding agencies in the public, commercial, or not-for-profit sectors.

### Competing interest statement

The authors declare no conflict of interest.

### Additional information

No additional information is available for this paper.
